# Exploring coronary microvascular dysfunction from functional impairment and structural damage

**DOI:** 10.3389/fcvm.2025.1600947

**Published:** 2026-01-29

**Authors:** Wei Wen, Yiqing Zhang, Genlin Jia, Yi Chi

**Affiliations:** 1 Department of Cardiovascular Diseases, Zhuhai Hospital of Integrated Traditional Chinese and Western Medicine, Zhuhai, China; 2 Department of Integrated Traditional Chinese and Western Medicine (Geriatrics Department), The People’s Hospital Medical Group of Xiangzhou, Zhuhai, China; 3 Department of Gastroenterology, Inner Mongolia Autonomous Region Hospital of Traditional Chinese Medicine, Hohhot, China

**Keywords:** coronary microvascular dysfunction, functional impairment, structural damage, ischemic heart disease, coronary flow reserve, index of microcirculatory resistance

## Abstract

Coronary microvascular dysfunction (CMD) is a syndrome characterized by myocardial ischemia resulting from structural and/or functional impairments of the coronary microvasculature, which includes pre-arterioles, arterioles, and capillaries. It has taken center stage in cardiovascular research due to its established role in triggering heart failure with preserved ejection fraction (HFpEF). The pathogenesis of CMD is closely associated with endothelial dysfunction, characterized by both structural and functional impairment of endothelial cells. This interplay between functional and structural injury underlies the significant heterogeneity in clinical phenotypes and hemodynamic characteristics across CMD subtypes, thus highlighting the necessity for a multidimensional investigation of its underlying pathological mechanisms. This review article systematically elaborates the pathophysiological features of CMD with a focus on two dimensions: microcirculatory functional regulation and vascular structural remodeling, aiming to provide a theoretical foundation for innovations in clinical diagnosis and treatment strategies.

## 1 Introduction

Ischemic heart disease (IHD) remains a leading cause of global mortality and disability. In clinical practice, the terms IHD and coronary artery disease (CAD) are often used interchangeably. However, a substantial proportion of patients with IHD exhibit non-obstructive coronary arteries, with stenosis <50% or even normal coronary anatomy. The ISCHEMIA (International Study of Comparative Health Effectiveness with Medical and Invasive Approaches) trial has challenged the traditional stenosis-centered therapeutic paradigm for IHD (1). As a major etiological component of IHD, coronary microvascular dysfunction (CMD)—defined as structural and/or functional abnormalities in the small coronary vessels (pre-arterioles, arterioles, and myocardial capillaries, typically <500 μm in diameter)—has garnered increasing attention. CMD manifests clinically as microvascular angina (MVA) with objective evidence of myocardial ischemia and is associated with an elevated risk of developing heart failure with preserved ejection fraction (HFpEF). Affected patients face a poor prognosis marked by recurrent hospitalizations, repeated invasive procedures, high rates of long-term adverse cardiovascular events, and a severely compromised quality of life. This syndrome thus represents a substantial public health challenge and contributes significantly to healthcare resource consumption.

## 2 Classification of pathological mechanisms of CMD

The coronary arterial system exhibits a characteristic tree-like branching architecture. Conventional coronary angiography visualizes only the epicardial vessels, which account for approximately 10% of the total coronary circulation, whereas the microvascular network (vessels <500 μm in diameter) comprises the remaining 90% of the vascular volume and serves as the critical determinant of myocardial perfusion (2). This anatomical structure fundamentally limits the diagnostic accuracy of traditional ischemia evaluation systems focused exclusively on epicardial stenosis in IHD. From a hemodynamic perspective, vascular resistance is inversely proportional to the fourth power of the vessel radius (per Poiseuille's law). This explains why large epicardial arteries function primarily as low-resistance conduits, contributing less than 5% of total coronary resistance, while the microcirculation—particularly vessels under 200 μm—regulates over 95% of coronary resistance (2, 5). Pre-arterioles (200–500 μm in diameter) respond dynamically to wall shear stress via endothelium-dependent mechanisms and contribute approximately 25% of total coronary resistance, playing a major role in regional blood flow distribution (2, 3). Pathological stimuli such as oxidative stress and inflammatory cytokines impair endothelium-dependent vasodilation, leading to abnormal pre-arteriolar constriction and significantly reduced coronary flow reserve (CFR) (4). This manifestation is recognized as functional microvascular dysfunction (FCMD). Clinically, endothelium-dependent CMD is characterized by normal epicardial arteries and reduced CFR, often provoked by acetylcholine challenge (4).

In contrast, arterioles (40–200 μm in diameter) regulate more than 50% of total coronary resistance through endothelium-independent mechanisms mediated by vascular smooth muscle cells (5, 6). Their dysfunction is frequently associated with structural remodeling. This zonal specialization of the microvasculature not only determines coronary flow regulation but also correlates with specific injury mechanisms: endothelial dysfunction predominantly affects pre-arterioles (endothelium-dependent dysfunction), whereas smooth muscle dysfunction mainly involves arterioles (endothelium-independent dysfunction).

Terminal microvessels (<40 μm in diameter) respond directly to myocardial metabolites (such as adenosine, H^+^, K^+^) to achieve precise matching of oxygen supply and demand. This hierarchical regulatory system exhibits adaptive coordination: under increased metabolic demand, metabolic vasodilation in terminal vessels reduces local resistance, thereby inducing myogenic relaxation in pre-arterioles, while arterioles coordinate upstream flow adaptation via endothelium-dependent pathways (5). Pathological disruption of endothelial–smooth muscle signaling cascades compromises this integrated regulation, leading to microvascular dysmotility and perfusion mismatch—the core pathophysiological basis of CMD.

The regulatory mechanisms of coronary microcirculation can be categorized into dynamic active regulation and static passive regulation: (1) Active regulation operates through neuroendocrine and paracrine signals to maintain microvascular tone homeostasis. Its core mechanism relies on metabolic-flow coupling. During increased myocardial oxygen demand, metabolites such as lactate and adenosine activate endothelial purinergic receptors (P1/P2Y) and transient receptor potential channels (TRPV4), leading to smooth muscle cell hyperpolarization and microvascular dilation. This process substantially augments coronary blood flow (CBF) (7). (2) Passive regulation, governed by hemodynamic and structural factors, involves coronary perfusion pressure gradients and vascular remodeling. Pre-arterioles sense changes in wall shear stress (WSS) and activate endothelium-dependent nitric oxide (NO) signaling pathways, enabling adaptive adjustments in perfusion pressure and flow (7). Notably, spatial heterogeneity in CBF exists across different segments of the left ventricular wall: microvascular resistance in the apical and mid-ventricular regions is 25%–40% higher than in basal regions (8). This anatomical characteristic renders the subendocardial myocardium particularly susceptible to ischemic injury. Under pathological conditions that elevate left ventricular wall stress (e.g., heart failure, dilated cardiomyopathy), mechanical compression of endocardial microvessels abruptly increases local resistance. This is compounded by compensatory elevation of resistance in upstream segments such as the left anterior descending artery (LAD), establishing a vicious cycle of “endocardial ischemia–microcirculatory deterioration” (9, 10). Distinct from the ischemic chest pain resulting from atherosclerotic narrowing of the epicardial coronary arteries, CMD originates from dysregulated coronary flow and a mismatch between myocardial oxygen supply and demand (11, 12). Its key pathophysiological mechanisms include enhanced microvascular contractility, diminished endothelium-dependent and endothelium-independent vasodilation, and increased microvascular resistance (5, 6). These alterations collectively lead to both functional and structural damage within the microvasculature.

### 2.1 Functional dysregulation

Functional dysregulation is characterized by impaired endothelium-dependent or endothelium-independent vasodilation and/or pathological vasoconstriction of coronary microvessels, mediated by inflammatory cytokines, adhesion molecules, oxidative stress, or autonomic nervous system dysregulation—all in the absence of structural microvascular damage (13, 14). Microvascular tone is finely regulated by vasodilators such as prostaglandins (15), NO, and endothelium-derived hyperpolarizing factors (EDHFs) (16), as well as vasoconstrictors like endothelin-1 (ET-1) (14, 17). Endothelium-derived NO serves as a key mediator of coronary vasodilation (18, 19), increasing the resting CBF, whereas ET-1 counteracts this effect through potent vasoconstriction and reduction in CBF (20, 21). The physiological balance between these pathways is essential for maintaining normal CBF, and its disruption underpins microcirculatory dysfunction.

The 2024 European Society of Cardiology (ESC) guidelines define FCMD by increased resting blood flow and an impaired hyperemic response (22). These guidelines further differentiate functional from structural (SCMD), noting that functional subtypes typically exhibit “higher resting flow,” while structural forms show a “blunted hyperemic response.” Clinical endotyping relies on invasive coronary function testing (ICFT) for accurate classification. Functional dysregulation exhibits a degree of reversibility and compensatory adaptation: microvascular dilation (with reduced resistance) may occur at rest, but during hyperemia, impaired endothelial-smooth muscle coupling severely restricts CFR, with peak flow reaching only 60%–80% of normal values (23). Persistently low microvascular tension promotes vascular stiffness—partly through reduced β-adrenergic sensitivity—accelerates the progression of HFpEF (24–28), and engages in a positive feedback loop with isolated arteriolar myogenic hyperreactivity (29).

### 2.2 Structural damage

**Figure 1:**
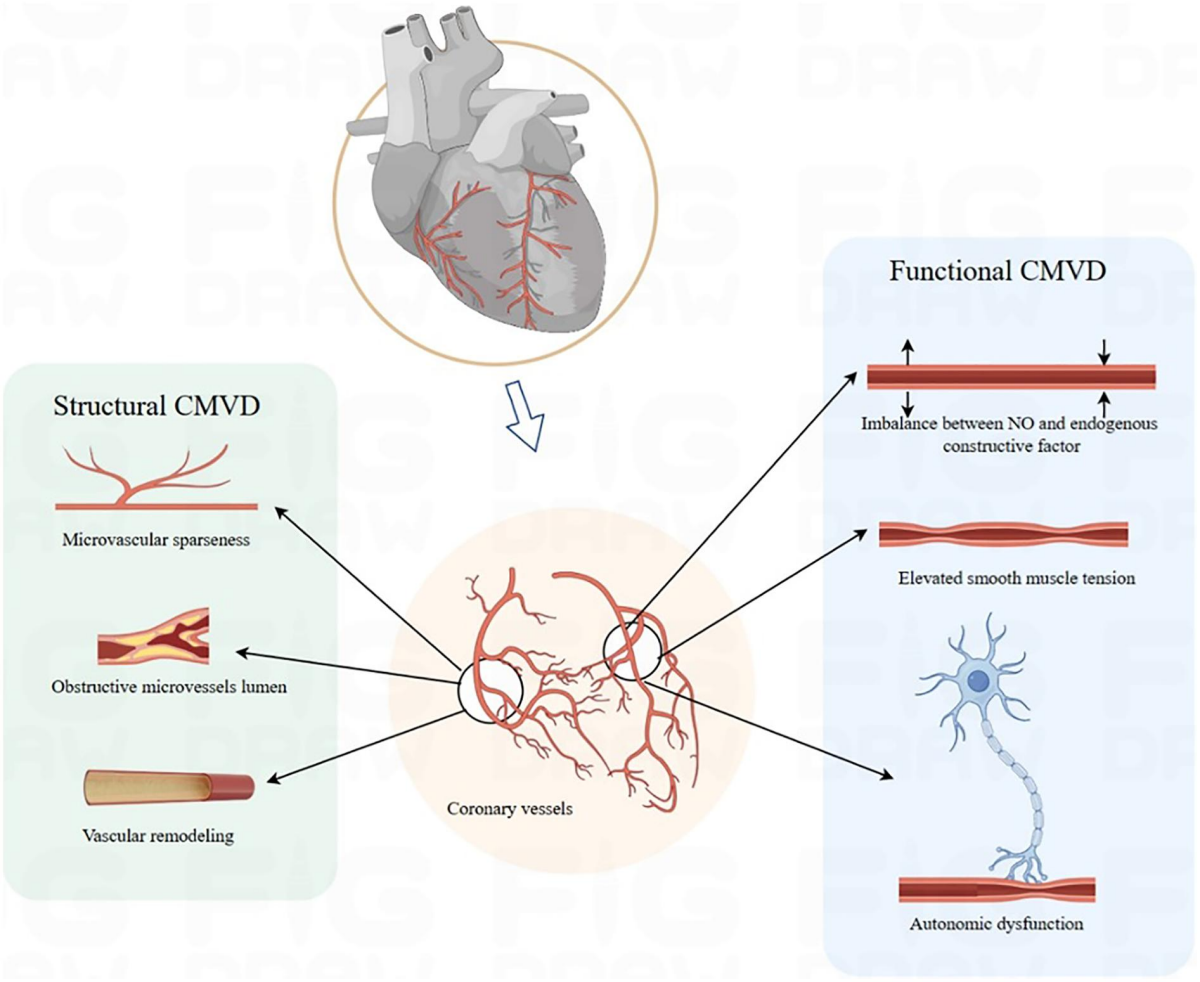
Pathophysiology mechanism. CMVD, coronary microvascular disease.

Structural damage in CMD encompasses microvascular stenosis, rarefaction, and luminal obstruction. Chronic exposure to inflammatory and oxidative stimuli induces endothelial hypertrophy and hyperplasia, which reduce lumen size and vascular compliance, ultimately leading to true microvascular stenosis (30). Studies demonstrate that CMD drives coronary microvascular remodeling primarily through smooth muscle cell proliferation—resulting in medial thickening—and perivascular fibrosis (31–33). Microvascular embolism may arise from distal embolization of microthrombi or platelet aggregates derived from ruptured epicardial plaques, often triggered by inflammatory mediators (34, 35). Reduced NO production by endothelial cells promotes collagen deposition and endothelial-to-mesenchymal transition (EndMT), which exacerbates microvascular rarefaction (36). These structural alterations also impair WSS-mediated activation of endothelial nitric oxide synthase (eNOS)—a mechanism that normally induces vasodilation—and thereby sustaining microvascular hyper-resistance (37). Consequently, structural damage often coexists with endothelium-dependent dysfunction, reflecting intertwined pathophysiological mechanisms. Endothelium-dependent CMD is correlated with accelerated atherosclerosis, increased plaque burden, and enhanced plaque vulnerability (38, 39). SCMD, characterized by narrowed luminal diameters or sparse vascular networks, causes persistently elevated resistance that restricts CBF below myocardial oxygen demand. In summary, FCMD is centered on reversible vasomotor dysregulation driven by an imbalance in endothelial-smooth muscle signaling, manifesting as dynamically elevated vascular resistance. Structural CMD, by contrast, results from fixed stenosis and vascular rarefaction, leading to persistently elevated resistance (Figure 1). These subtypes frequently coexist and interact: chronic endothelial dysfunction in FCMD promotes smooth muscle proliferation and collagen deposition, thereby accelerating microvascular remodeling. Conversely, structural stenosis forces compensatory dilation in residual microvessels, exacerbating endothelial injury and oxidative stress and worsening functional impairment.

## 3 Clinical characteristics of CMD subtypes

### 3.1 Definition

CMD is a clinical syndrome resulting from structural or functional abnormalities in pre-arterioles and arterioles, pathologically manifesting as elevated microvascular resistance and impaired vasodilatory capacity. This leads to exertional angina or objective evidence of myocardial ischemia (40). Based on patterns of endothelial injury, CMD—characterized by reduced CFR—is classified into two endotypes: functional coronary microvascular dysfunction (FCMD) and structural coronary microvascular dysfunction (SCMD) (41, 42). Specifically, FCMD is defined by reduced CFR, reversibly elevated index of microcirculatory resistance (IMR), and low resting microvascular resistance, reflecting impaired vasodilatory efficiency due to endothelial hyperreactivity. In contrast, SCMD presents with both reduced CFR and persistently elevated IMR, indicating fixed high resistance resulting from organic stenosis (43). Both subtypes compromise coronary perfusion efficiency and impair the regulation of coronary blood flow. As highlighted by Haseeb Rahman et al., patients with CMD exhibit significantly reduced hyperemic myocardial perfusion efficiency compared to controls (controls: 61% ± 12%; FCMD: 44% ± 10%; SCMD: 42% ± 11%, *P* < 0.001), with SCMD demonstrating greater differences in IMR than FCMD (*Δ*IMR 2.7 ± 0.8 vs. 1.5 ± 0.6 U) (42). These findings may be linked to progressive attenuation of endothelium-dependent dilation in FCMD. Hemodynamic profiling further differentiates two phenotypes: low-resistance CMD (LHR-CMD), marked by reduced resting microvascular resistance (IMRrest 34.3 ± 15.1 U, *P* < 0.01) and accelerated resting flow (Tmnrest 0.37 ± 0.17, *P* < 0.01), and high-resistance CMD (HHR-CMD), characterized by limited hyperemic flow (Tmnhyp 0.45 ± 0.24, *P* < 0.01) (44). Rahman's study suggests that SCMD may exhibit larger gradients between resting and hyperemic resistance, t though this remains unvalidated due to incomplete disclosure of data (42). This underscores the potential of dual-state (resting/hyperemic) hemodynamic analysis as a future diagnostic tool.

### 3.2 Epidemiology

Large-scale population studies on the prevalence of CMD remain limited. The AID-ANGIO study (Advanced Invasive Diagnosis-ANGIOgraphy), which included 317 patients with chronic coronary syndrome (CCS), reported a 45.1% prevalence of ischemia with no obstructive coronary arteries (INOCA) (45). The WISE (Women's Ischemia Syndrome Evaluation) study identified CMD in 39% of enrolled women (46), whereas the iPOWER study (ImProve diagnOsis and treatment of Women with angina pEctoris and micRovessel disease) reported a lower prevalence of 26% (47). According to the BELmicro registry (Belgian Registry on Coronary Function Testing), CMD was diagnosed in 23.4% of INOCA patients (48).

These discrepancies likely reflect heterogeneity in diagnostic criteria and thresholds across studies. A meta-analysis indicated that non-invasive diagnostic strategies yielded a higher pooled prevalence of CMD (43%) compared to invasive methods (28%) (49), though current clinical guidelines estimate that CMD affects 45%–60% of INOCA patients (50). Subtype distribution also varies considerably among studies: some report a predominance of FCMD (e.g., 62% FCMD vs. 38% SCMD) (42), while others report FCMD in 60.8% and SCMD in 39.2% of cases (41). In contrast, a study by David Hong et al. involving 375 patients found a higher prevalence of SCMD (45.0%, defined as preserved CFR with elevated IMR) compared with FCMD (33.9%, defined as preserved CFR with low IMR) (Table 1) (51). These inconsistencies underscore the diagnostic challenges arising from the lack of standardized criteria. Most studies rely on non-invasive imaging without concomitant invasive IMR measurements, highlighting the urgent need for a unified diagnostic framework that integrates dual-state (resting and hyperemic) hemodynamic parameters.

**Table 1:** Prevalence of CMD.

Studies/author	Study type	Study population	Sample size	Diagnostic tool	Prevalence rate	References
AID-ANGIO study	Prospective study	CCS	260	Pressure guide wire (IMR)	45.1%	46
WISE study	Prospective study	CCS	210	Doppler-tipped guidewire (CFR)	39%	47
iPOWER study	Prospective study	Non-CAD	919	Transthoracic Doppler echocardiography (CFR)	26%	48
BELmicro	Prospective study	Non-CAD	449	Unknown	23.4%	49
Aribas E	systematic review	Unknown	4,42,206	Intrusive strategy and non-intrusive strategy	43% non-invasive; 28% invasive	51
Rahman H	Prospective study	Non-CAD	375	Pressure guide wire (IMR)	FCMD 60.8%, SCMD 39.2%	52
Hong D	cross-sectional study	Non-CAD	86	Doppler-tipped guidewire (CFR)	53.5%	53

AID-ANGIO study, advanced invasive diagnosis for patients with chronic coronary syndromes undergoing coronary angiography study; CCS, chronic coronary syndrome; WISE study: women's ischemia syndrome evaluation study; iPOWER study, ImProve diagnOsis and treatment of Women with angina pEctoris and micRovessel disease study; CAD, coronary artery disease; BELmicro registry, (Belgian Registry on Coronary Function Testing); FCMD, functional coronary microvascular dysfunction; SCMD, structural coronary microvascular dysfunction.

### 3.3 Clinical manifestations

CMD is frequently misdiagnosed as cardiac neurosis due to the lack of significant coronary stenosis (52). Although symptoms may overlap with those of obstructive CAD, patients with INOCA are at higher risk for CMD (53). The most common clinical presentation is angina pectoris, typically occurring upon exertion and sometimes at rest. However, these symptoms can be non-specific, often leading to underrecognition or misdiagnosis. Clarifying these manifestations is essential for bridging the pathophysiological mechanisms of CMD to clinical practice, prompting clinicians to include CMD in the differential diagnosis of patients with unexplained myocardial ischemia.

In HFpEF, numerous studies indicate that at least 50% of patients have concomitant CMD. As highlighted by Mohammed et al. (54), CMD is independently associated with adverse outcomes in HFpEF, including increased heart failure readmissions and elevated all-cause mortality. A meta-analysis involving 1,138 HFpEF patients reported an overall CMD prevalence of 58%. CMD was associated with more severe echocardiographic diastolic dysfunction, higher prevalence of atrial fibrillation (OR = 1.61), and a doubled risk of death or heart failure hospitalization (OR = 3.19) (55). Furthermore, the PROMIS-HFpEF study, which enrolled 202 HFpEF patients without significant epicardial CAD, found a CMD prevalence of approximately 75% (defined as CFR < 2.5). CMD was associated with systemic endothelial dysfunction (lower Endothelial Pulse Amplitude Tonometry/ Reactive Hyperemia Index), higher urinary albumin-to-creatinine ratio and N-terminal pro-brain natriuretic peptide levels, and impaired right ventricular mechanics (56). These findings are largely attributed to chronic microvascular hypoperfusion and progressive myocardial fibrosis, underscoring the importance of CMD assessment in the clinical management of HFpEF. In advanced stages, CMD often progresses to HFpEF. Accurate subtyping of CMD in HFpEF (using CFR and IMR/HMR thresholds) is critical for guiding treatment. Non-invasive or invasive measurement of CFR confirms CMD diagnosis, while IMR < 25 U and HMR < 2.5 mmHg·s/cm indicate FCMD, and IMR ≥ 25 U or HMR ≥ 2.5 mmHg·s/cm indicate SCMD (22). Regular reassessment of symptoms and tailored escalation of therapy are essential for effective long-term management.

In myocardial infarction with non-obstructive coronary arteries (MINOCA), CMD is a key and frequent pathological mechanism. CMD—particularly functional phenotypes such as microvascular spasm or endothelial dysfunction—is recognized as a key trigger for MINOCA (57). Microvascular spasm is not only linked to rest angina but may also precipitate acute coronary events presenting as MINOCA, emphasizing the utility of functional assessments (e.g., acetylcholine provocation testing) to identify treatable CMD endotypes. A notable proportion of patients with MINOCA coexist with CMD; comprehensive assessment of CMD confers significant prognostic value in MINOCA, as it facilitates the identification of high-risk subgroups and guides personalized management to improve clinical outcomes. Additionally, accurate and timely diagnosis of CMD is crucial for optimizing prognostic stratification and clinical management in patients with MINOCA (57).

Within the INOCA population, CMD serves as the principal mechanism underlying myocardial ischemia and often manifests as primary MVA. Data from the WISE study confirm that INOCA patients face poor clinical outcomes, with reduced CFR—a hallmark of CMD—serving as an independent predictor of major adverse cardiovascular events (MACE) (58). Thus, CMD evaluation is critical for risk stratification and treatment personalization in INOCA, especially since FCMD phenotypes may respond to targeted therapies such Angiotensin-Converting Enzyme inhibitors (ACEI)/Angiotensin Receptor Blockers (ARB).

In CCS, CMD frequently coexists with obstructive CAD. This comorbidity contributes to ischemia in territories supplied by non-stenotic arteries and synergistically worsens myocardial perfusion in areas distal to epicardial narrowings. Chronic adaptive changes in the microvasculature under persistent hypoperfusion may lead to structural remodeling and impaired maximal vasodilation—a phenotype of SCMD (59). Notably, the presence of CMD can cause fractional flow reserve (FFR) to underestimate the functional significance of epicardial lesions, explaining frequent mismatches between angiographic stenosis severity and ischemic burden. This underscores the clinical value of combining FFR with invasive microvascular function testing in patients with CCS.

### 3.4 Clinical differentiation

Most patients with CMD present with exertional angina (60), while a minority report exertional dyspnea, reflecting variations in microvascular impairment patterns. In FCMD, ischemia is often masked at rest due to paradoxical microvascular dilation, becoming symptomatic only during exertion as a result of insufficient CBF. In contrast, SCMD is characterized by persistently elevated microvascular resistance at rest (IMR >25 U), leading to perfusion deficits even under low metabolic demand—a finding consistent with those reported by Rahman et al. (42).

Gender differences are also evident: although females are less commonly affected by obstructive CAD, they represent a higher proportion of non-obstructive CAD and CMD cohorts (61, 62). While one study by Hong et al. reported no gender differences between CMD subtypes (with 65.8% female representation in both FCMD and SCMD) (51), FCMD may disproportionately affect women, potentially due to autonomic dysregulation linked to emotional stress (63), although this association requires further investigation.

Regarding risk factors and prognosis, patients with SCMD show higher prevalence of hypertension and diabetes compared to those with FCMD, although exercise capacity and left ventricular ejection fraction are generally comparable between the two groups (42). Pharmacological testing reveals that SCMD patients exhibit a delayed forearm blood flow response to acetylcholine compared to FCMD (2.1 ± 1.8 vs. 4.1 ± 1.7, *P* = 0.001) (64), suggesting that structural remodeling contributes to impaired endothelial-smooth muscle signaling.

Prognostically, CMD is not a benign condition. A meta-analysis indicated that CMD is associated with a 3.9-fold increase in all-cause mortality and a 5.2-fold higher risk of MACE compared to normal microvascular function (65). Impaired CFR is strongly correlated with adverse outcomes (66). Specifically, SCMD is linked to higher mortality, whereas FCMD is associated with increased MACE risk (41). Elevated IMR also predicts disease progression, with each unit increase raising MACE risk by 5% (33). Similarly, Toya et al. reported a 0.7-fold increase in MACE risk per unit reduction in CFR (67), suggesting that SCMD may portend a worse prognosis. These findings highlight the prognostic importance of assessing minimal microvascular resistance (68).

**Figure 2:**
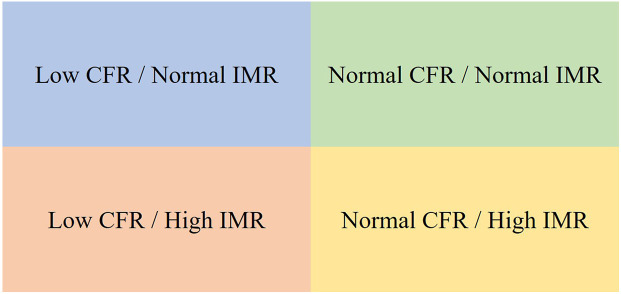
Prognostic stratification by CFR and IMR. ▪ No significant MACE/mortality risk. ▪ Increased MACE risk but no significant change in mortality. ▪ Both MACE and mortality risk are high. ▪ Lower MACE and mortality risk. CFR, coronary flow reserve; IMR, index of microcirculatory resistance; MACE, adverse cardiovascular events.

It is essential to keep the CFR–IMR relationship precise: low CFR is the dominant signal for CMD diagnosis; low CFR and high IMR carries the highest risk. However, evidence remains nuanced. Data from the ILIAS registry indicate that a low CFR (≤2.5) is the dominant prognostic indicator for MACE, whereas an elevated IMR alone lacks independent prognostic value. Moreover, among patients with low CFR, both functional (low CFR/normal IMR) and structural (low CFR/high IMR) endotypes demonstrated similar event rates (41). Similarly, a network meta-analysis showed that the combination of low CFR and high IMR was associated with the highest risk of MACE and mortality. Isolated low CFR with normal IMR correlated with increased MACE but neutral mortality risk, and isolated high IMR with normal CFR did not significantly increase risk compared to patients with normal microvascular function (69) (Figure 2).

## 4 Diagnosis of CMD

The diagnosis of CMD relies on a combination of imaging and functional assessments, which can be broadly categorized into invasive and non-invasive techniques (22, 50). Non-invasive approaches primarily include myocardial perfusion imaging (MPI), single-photon emission computed tomography (SPECT), and cardiac magnetic resonance imaging (CMR) (70). These methods typically involve the administration of peripheral vasodilators followed by observation of myocardial perfusion using contrast agents (summarized in Table 2). Transthoracic Doppler echocardiography (TTDE) is limited to assessing flow velocity in the left anterior descending artery (LAD) (71), making comprehensive non-invasive strategies more suitable for evaluating overall myocardial perfusion efficacy.

**Table 2:** Diagnostic strategy.

Modality	Parameter	Diagnostic threshold	Technique	Superiority	Inferiority
Non-invasive techniques					
transthoracic Doppler echocardiography	CFR	CFR < 2.0	Pulsed-wave Doppler on the proximal LAD artery	Time saving; Low cost; diagnosis Repeatable measurement; Inspect at the bedside.	Limited to LAD region; Training needed
Positron Emission Tomography	MBF	MBF < 2	Quantification of blood flow per gram of myocardium per minute	Gold standard for non-invasive assessment of coronary microvascular function; Overall assessment of whole-heart and local myocardial microvascular function.	Limited spatial resolution; High costs; Obstructive CAD need to be excluded
Cardiac Magnetic Resonance	MPR	MPR > 2.25	Dynamic first-pass vasodilator stress and then rest perfusion imaging	High spatial resolution; Accurate evaluation of endocardial and subepicardial myocardial perfusion; Coronary artery resistance and diastolic filling time	Gadolinium contrast agents cause adverse reactions in patients with renal insufficiency
Dynamic Myocardial Perfusion CT	MBF	MBF < 2	Dynamic first-pass vasodilator stress and then rest perfusion imaging	High spatial resolution; Allows anatomical and functional assessment of the myocardium and coronary circulation in a single examination	Higher radioactivity; Low accuracy; High heart rate and coronary artery calcification may reduce image quality and may cause adverse effects in patients with renal insufficiency
Myocardial contrast echocardiography	CFR	CFR < 2	Myocardial blood flow and blood flow under resting and load conditions	No radioactive damage Real-time and repeated measurements at the bedside; Cheaper than other non-invasive tests	Changes in the concentration of microbubbles and interference with the inhomogeneity of ultrasonic power; Contrast-associated complications
Modality	Parameter	Diagnostic Threshold	Technique	Superiority	Inferiority
Invasive techniques					
Intracoronary temperature-pressure wire	IMR; FFR	CFR <2–2.5; FFR >0.8 IMR >25 U; RRR <2.62 (91)	Estimate of coronary blood flow; or continuous thermodilution techniques	Small degree of discrepancy; High reliability; IMR: unaffected by hemodynamics; High specificity; High accuracy.	Invasive tests; Require rapid injection of high doses of coronary artery dilators such as adenosine.
Intracoronary Doppler flow-pressure wire	HMR CFVR	CFVR < 2.5 HMR > 2.5 mmHg·s/cm (85)	Direct measurement of coronary peak flow velocity	Combined assessment microvascular dysfunction; unaffected by hemodynamics; Classification according to HMR involving SCMD and function CMD	Invasive tests; Ununified cut-off values of HMR
Angiography-derived index of microcirculatory resistance (Angio-IMR)	Angio-IMR QFR	QFR > 0.8; Angio-IMR>25 U	Transformation of hydrodynamics derived angiography	Simple procedure; No pressure guidewire; No vasodilator drugs; No additional surgical operations	Ununified cut-off values of HMR; Unknown the accuracy of evaluating CMD; Affected by image quality

Thresholds/cut-offs (standardize): Invasive CFR: <2.5; FFR: >0.80; IMR: ≥25 U; HMR: >2.5 mmHg·s/cm; QFR: >0.80.

CFR, coronary flow reserve; CMD, represents coronary microcirculation dysfunction; LAD, represents left anterior descending branch; MBF, myocardial blood flow; MPR, myocardial perfusion reserve; MPR, myocardial perfusion reserve; IMR, represents index of microcirculation resistance; FFR, represents fractional flow reserve; HMR, represents hyperemic microvascular resistance; RRR, resistance reserve ratio; CFVR, coronary flow velocity reserve.

**Figure 3:**
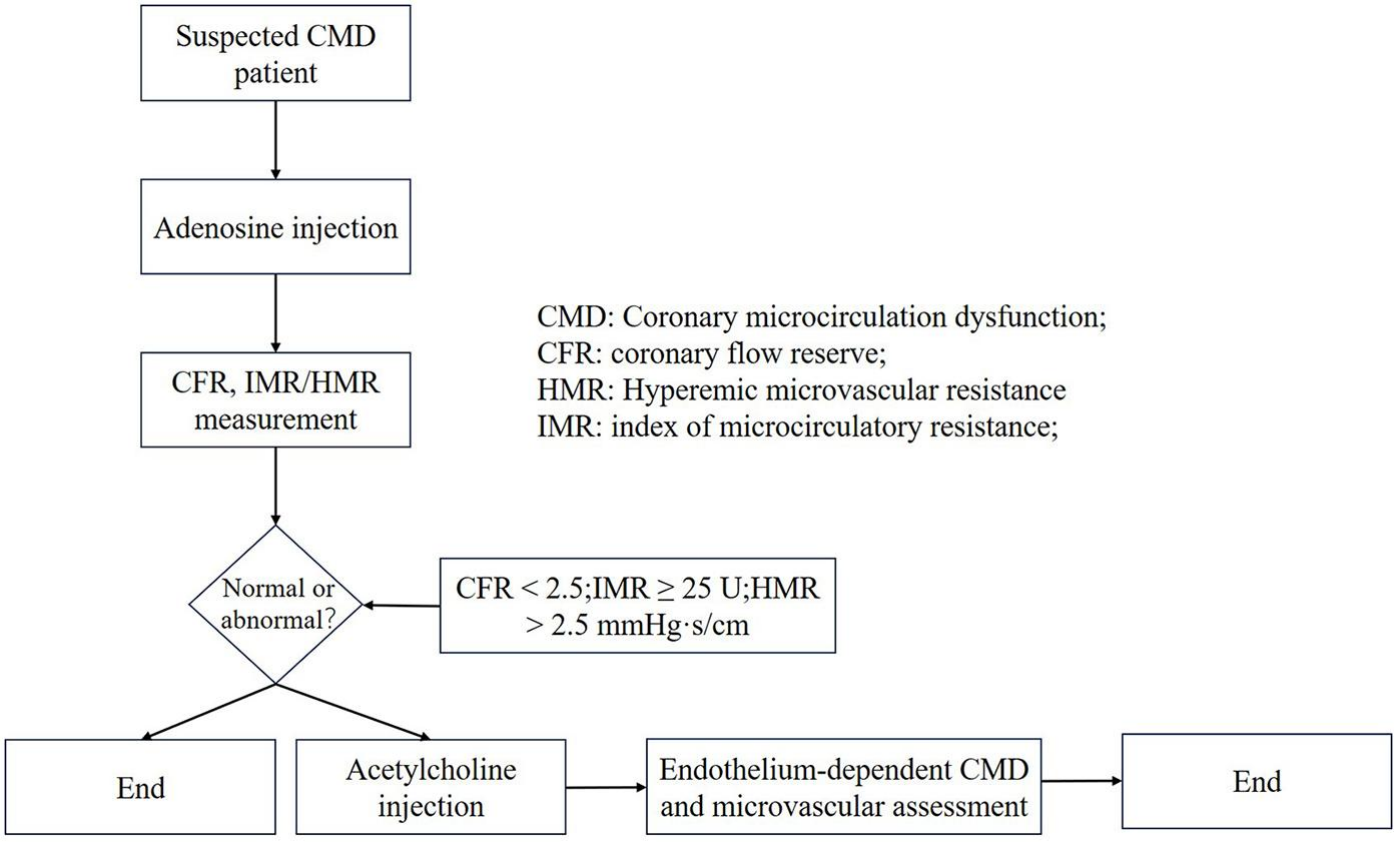
Invasive coronary function testing. CMD, coronary microcircualation dysfunction; CFR, coronary flow reserve; HMR, hyperemic microvascular resistance; IMR, index of microcirculatory resistance.

Invasive strategies employ coronary functional indices such as FFR, CFR, and the IMR. Diagnostic thresholds for CMD vary depending on the assessment modality (e.g., PET, CMR, thermodilution, or Doppler), with a commonly accepted clinical range of CFR <2.0–2.5. Specifically, a thermodilution-derived CFR <2.0 has shown low diagnostic sensitivity, whereas adopting a threshold of CFR <2.5 (consistent with Doppler-based criteria) yields more reasonable accuracy (72–74). To standardize the application of ICFT for CMD diagnosis (Figure 3), a concise algorithm is recommended: begin with the adenosine phase [to assess CFR and IMR/hyperemic microvascular resistance (HMR)], followed by the acetylcholine (ACh) phase (to evaluate endothelium-dependent function and test for epicardial or microvascular spasm). Diagnostic thresholds are defined as CFR <2.5, IMR ≥25 U, and HMR >2.5 mmHg·s/cm. It should be emphasized that coronary spasm may occur in the presence of normal baseline CFR and microvascular resistance values; therefore, ACh provocation testing is essential for its diagnosis.

### 4.1 FFR

FFR is defined as the ratio of distal coronary pressure (Pd) to proximal aortic pressure (Pa) under conditions of maximal hyperemia. Introduced by Nico H.J. Pijls to estimate coronary flow using pressure-derived measurements (75), FFR reflects the functional significance of epicardial stenosis by quantifying the relationship between myocardial blood flow and perfusion pressure during hyperemia. The DEFER trial (76) and current ESC guidelines (Class IA recommendation) (77) have established the critical role of FFR in guiding percutaneous coronary intervention (PCI). While FFR primarily evaluates epicardial stenosis, a value >0.80 effectively excludes ischemia caused by epicardial vessels, thereby facilitating subsequent assessment of microvascular function using complementary indices.

### 4.2 CFR

First conceptualized in 1974, CFR is defined as the ratio of hyperemic to resting coronary flow velocity (74). Although it cannot localize the level of impairment (epicardial vs. microvascular), CFR provides a comprehensive assessment of ischemic contributions from both compartments. In the absence of epicardial stenosis, a reduced CFR (<2.0) is widely accepted as indicative of CMD (74), though some studies support a threshold of <2.5 (78), reflecting methodological and population variability.

### 4.3 IMR

In 2003, Professor William F. Fearon introduced IMR as a specific measure of microvascular resistance and validated it in animal models, demonstrating strong correlation with true microcirculatory resistance (79). Subsequent clinical studies confirmed that IMR exhibits high consistency with actual microvascular resistance, characterized by low intrinsic variability, minimal hemodynamic influence, high reproducibility, and independence from epicardial stenosis (80). In 2016, Professor D. Carrick et al. (81) analyzed data from 288 PCI patients and demonstrated a significant correlation between IMR and CFR. IMR is mathematically defined as the product of Pd and the mean transit time (Tmn) of a saline bolus under hyperemic conditions:

IMR = Pd × Tmn (Pd = distal coronary pressure; Tmn = mean transit time); Chufan Luo et al. (82) established a normal IMR reference range of 13.2–22.4 U in healthy individuals. Melikian et al. (83) proposed a diagnostic threshold of IMR >25 U for CMD, based on a study involving 101 CAD patients and 15 controls. Roberto Scarsini et al. (84) further suggested thresholds of IMR >40 U for ST-Elevated Myocardial Infarction (STEMI) patients and IMR >25 U for those with Non-ST-Segment Elevation Myocardial Infarction (NSTEMI) or CCS.

### 4.4 HMR

HMR is defined as the ratio of Pd to mean hyperemic flow velocity. Diagnostic thresholds for HMR vary across studies: some have used HMR ≥2.0 mmHg·s/cm (85), while others propose a cutoff of ≥2.5 mmHg·cm^−^¹·s (86). HMR has been shown to moderately correlate with IMR and may offer advantages in predicting microvascular functional impairment (87).

The diagnostic framework for CMD is based on a multiparametric functional assessment system. Current guidelines recommend using CFR <2.0 as a core criterion, combined with IMR >25 U (mmHg·s) or HMR ≥2.5 mmHg·s/cm to define SCMD. FCMD is characterized by reduced CFR (<2.5) with low resting resistance (IMR <25 U or HMR <2.5 mmHg·s/cm) (41). These criteria align with the pathophysiological features of resting hyperperfusion in FCMD and elevated hyperemic resistance in SCMD (41, 42).

The microvascular resistance reserve (MRR) has emerged as a novel stratification tool. FCMD typically exhibits reduced resting microvascular resistance and accelerated baseline flow, whereas SCMD demonstrates limited hyperemic flow. However, no significant difference in MRR has been observed between CMD subtypes (*P* = 0.66) (44). An MRR <2.7 is currently regarded as diagnostic for CMD (88). Clinical studies indicate that abnormal MRR is associated with increased risks of cardiovascular death (RR = 4.88), MACE (RR = 2.37), and myocardial infarction (RR = 1.93), with lower values correlating with poorer survival (89). However, further prospective studies and randomized controlled trials are needed to validate these associations.

The resistance reserve ratio (RRR), defined as the ratio of baseline to hyperemic microvascular resistance, directly reflects microvascular responsiveness to vasodilatory stimuli. RRR is less influenced by epicardial stenosis and hemodynamic variability than CFR (90). Studies show that INOCA patients with RRR <2.62 face a 1.6-fold increased mortality risk (91). Theoretically, MRR may help detect larger resistance changes (*Δ*IMR) in SCMD, though clinical validations are still lacking. Other invasive methods, such as exercise stress testing (92) and TIMI frame count (93), remain controversial for definitive CMD diagnosis.

**Figure 4:**
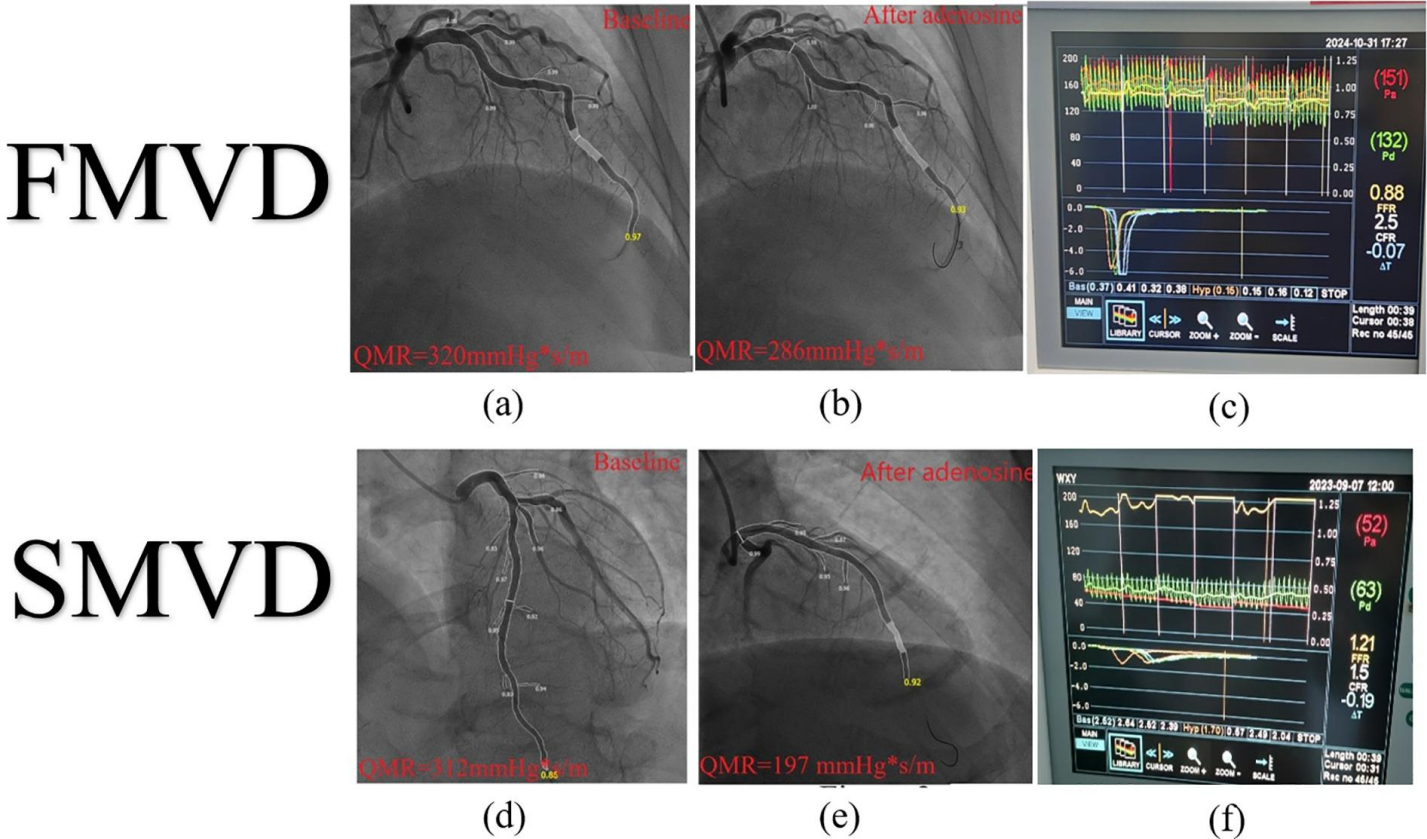
IMR and QMR. IMR, index of microcirculatory resistance; QMR, quantitative microvascular resistance; SCMVD, structural coronary microvascular disease; FCMVD, functional coronary microvascular disease. FCMVD displays the patient's IMR: 132 × 0.15–19.8 U, CFR = 2.5 **(c)** The difference between QMR baseline and hyperemic state is:34 mmHg*S/m **(a,b)**; SCMVD displays the patient's IMR: 63 × 1.7–107.1 U, CFR = 1.5 **(f)** The difference between QMR baseline and hyperemic state is:115 mmHg*S/m **(d,e)**.

To address practical challenges in IMR measurement (e.g., equipment cost and procedural duration), angiography-derived indices such as quantitative microvascular resistance (QMR) (94) and coronary angiography-derived IMR (CaIMR) (95) have been developed. A meta-analysis of 15 studies reported that Angiography-derived IMR exhibits high diagnostic accuracy for CMD (sensitivity 0.84, specificity 0.87, AUC = 0.91) (96). Preliminary observations from our ongoing AWARD study (Aromatic and Warming-Up Management in Coronary Microvascular Disease) (97) indicate that SCMD patients (meeting both CFR <2.5 and IMR >25 U) demonstrate greater differences in QMR between resting and hyperemic states compared to FCMD patients (Figure 4), providing visual evidence for subtype differentiation.

## 5 Management of CMD

The increasing recognition of ischemic chest pain related to CMD parallels advancements in coronary intervention techniques and functional assessment modalities. Substantial evidence confirms that CMD is an independent risk factor for MACE, highlighting the critical importance of early intervention to improve prognosis. As emphasized by William E. Boden (98), therapeutic strategies should prioritize reducing MACEs to enhance clinical outcomes while improving patients’ quality of life.

### 5.1 Risk factor control

Although CMD is a non-atherosclerotic disorder, ample evidence indicates that traditional atherosclerotic risk factors significantly contribute to microvascular endothelial injury and disease progression. The COURAGE trial (99) established that antiplatelet therapy combined with intensive lipid-lowering (statins), glycemic control, and Renin-Angiotensin-Aldosterone System (RAAS) inhibition forms a cornerstone strategy for preventing cardiovascular events. The ISCHEMIA trial further supports the incorporation of PCSK9 inhibitors into the management of ischemic heart disease (100). Clinical protocols should include dynamic monitoring of blood pressure, lipids, and glucose levels tailored to individual risk profiles, along with exercise training to improve cardiopulmonary reserve and endothelial repair capacity (101). Yuting Han et al. (102) reinforce that comprehensive lifestyle interventions—including dietary modification and regular physical activity—should form the foundation of CMD management.

### 5.2 Anti-anginal therapy

The WARRIOR trial evaluated intensive medical therapy (statins, ACEI/ARB, and aspirin) combined with risk factor control in women with suspected INOCA-related CMD (not ICFT-confirmed). At 5-year follow-up, no significant difference in MACEs was observed between the intensive therapy and usual care groups; brief methodological caveats for this neutral outcome include insufficient statistical power, heterogeneity in CMD mechanisms, low event rates, and issues related to treatment adherence and crossover (103). By contrast, the CorMicA trial centered on ICFT-guided therapy—a key distinction from WARRIOR's non-ICFT-targeted approach—and demonstrated that this mechanism-specific intervention yielded meaningful improvements in angina symptoms and patient-reported quality of life (QoL) for CMD patients. This finding underscores the contrast between CorMicA's clear symptom/QoL benefits with ICFT guidance and WARRIOR's neutral MACE results when comparing intensive vs. usual care (104). Ongoing trials such as ENDOFIND (105) and CorCTCA (106) are evaluating endothelial function modulation and precision medicine approaches for INOCA subtypes. Registry data from POL-MKW indicate that patients with abnormal IMR/CFR are more frequently prescribed calcium channel blockers (CCB), ACEIs (107), and trimetazidine (118). The ChaMP-CMD trial demonstrated that amlodipine and ranolazine significantly improved exercise duration in CMD patients (108). Other agents including ivabradine, fasudil, and Sodium-Glucose Co-Transporter 2 (SGLT2) inhibitors also show therapeutic potential. Current guidelines recommend prioritizing risk factor control, with symptom relief strategies followed by β-blockers, CCBs, and nicorandil (7, 22). For refractory CMD cases, the Coronary Sinus Reducer (CSR) may be considered only in highly selected patients, typically within specialist centres or registries. While CSR has shown signals of improving angina and quality of life (QoL), its benefit on hard outcomes remains unproven (109).

### 5.3 Endothelial function modulation

Given the central role of endothelial dysfunction in CMD pathogenesis, targeting endothelial repair represents a promising therapeutic strategy. ACEIs improve endothelial function by inhibiting RAAS-mediated injury and enhancing NO bioavailability (110). The IMPROvE-CED trial confirmed that intracoronary infusion of CD^34+^ cells significantly increases microvascular flow velocity, offering a novel regenerative approach (111). The PRIZE study demonstrated that zibotentan, an endothelin antagonist, effectively reverses endothelial dysfunction in microvascular angina patients carrying specific ET-1 variants, highlighting the potential of genotype-guided therapy (112). Mesenchymal stem cell-based therapies, such as those evaluated in the CHART-1 trial, promote myocardial reverse remodeling and functional improvement (113), though larger trials are needed to confirm their efficacy.

### 5.4 Personalized stratified therapy

Stratified treatment approaches represent a shift toward precision medicine in CMD. The CorMicA study introduced the concept of “functional angiography” to guide pharmacotherapy based on endotype (104). Current evidence supports classifying CMD into FCMD and SCMD subtypes, each requiring distinct management strategies.

FCMD is primarily characterized by endothelial dysfunction and abnormal vasoconstriction. Treatment for this endotype should focus on endothelial improvement and metabolic optimization, including graded exercise to enhance coronary flow autoregulation (114), emotional management to avoid autonomic dysregulation, and medications such as ACEI/ARBs, nicorandil (115), and trimetazidine (116). For FCMD specifically, high-dose β-blockers are generally not recommended, as they may exacerbate abnormal vasoconstriction in this subtype.

SCMD involves structural microvascular damage, and treatment for this endotype requires therapies to inhibit remodeling and fibrosis. Statins are first-line agents for SCMD to modify microvascular structure (117), complemented by diuretics to reduce ventricular preload and endothelin antagonists like zibotentan for endothelial repair (112, 119).

## 6 Conclusion

CMD is pathologically stratified into FCMD and SCMD subtypes based on distinct endothelial dysfunction patterns and microvascular functional/structural features (Table 3). Although research in this area continues to evolve, these subtypes are characterized by divergent pathophysiological mechanisms, clinical presentations, and prognostic profiles, underscoring the need for precision therapeutic approaches. Current priorities in the field include the development of novel endothelial-targeted treatments with demonstrated translational potential. Nevertheless, guideline recommendations for subtype-specific management still require validation through large-scale clinical trials to optimize evidence-based care and improve patient outcomes.

**Table 3:** Features of FCMD vs. SCMD.

Type of CMD	Pathological characteristics	Microcirculatory manifestations	Diagnosis	Progress	Clinical manifestations	Management
FCMD	Impaired endothelium-dependent or -independent vasodilation and/or pathological vasoconstriction	Reversibility: compensatory microvascular dilation (reduced resistance) occurs at rest, but impaired endothelial-smooth muscle coupling during hyperemia severely limits CFR.	Decreased CFR and Near normal IMR	Developing HFpEF and MACE	Maybe more high prevalence; exertional angina	Improving endothelial function, optimizing myocardial metabolism, increasing exercise equivalent appropriately, and emotional management,
SCMD	Microvascular stenosis, rarefaction, and luminal obstruction	Incomplete reversibility; microvascular remodeling causing persistently elevated resistance that restricts CBF.	Decreased CFR and increased IMR	Poor prognosis; developing mortality.	Resting angina, combined higher prevalence s of hypertension, diabetes	Anti-remodeling agents, antifibrotics, and afterload reduction, alongside endothelial repair

FCMD, functional coronary microvascular dysfunction; SCMD, structural coronary microvascular dysfunction; CFR, coronary flow reserve; IMR, index of microcirculatory resistance; HFpEF, heart failure with preserved ejection fraction; MACE, major adverse cardiovascular events; CBF, coronary blood flow.
